# Effect of Selenium Supplementation on Glutathione Peroxidase Enzyme Activity in Patients With Chronic Kidney Disease: A Randomized Clinical Trial

**DOI:** 10.5812/numonthly.17945

**Published:** 2014-05-04

**Authors:** Omid Sedighi, Mehryar Zargari, Gharmohammad Varshi

**Affiliations:** 1 Department of Nephrology, Faculty of Medicine, Imam Khomeini Hospital, Mazandaran University of Medical Sciences, Sari, IR Iran; 2 Department of Clinical Biochemistry and Genetics, Faculty of Medicine, Mazandaran University of Medical Sciences, Sari, IR Iran; 3 Department of Internal Medicine, Imam Khomeini Hospital, Mazandaran University of Medical Sciences, Sari, IR Iran

**Keywords:** Renal Insufficiency, Chronic, Glutathione Peroxidase, Selenium

## Abstract

**Background:**

Plasma selenium (Se) concentration and glutathione peroxidase (GSH-Pxs) enzyme activity of the patients with chronic kidney disease (CKD) are usually lower than healthy individuals; however, the effect of Se supplementation on the GSH-Pxs activity in those patients remains unclear.

**Objectives:**

This study aimed to assess the effect of Se supplementation on plasma Se concentration and red blood cell (RBC) GSH-Pxs activity in patients with different stages of CKD.

**Patients and Methods:**

In this randomized clinical trial, forty-five patients with CKD who attended in a nephrology clinic were recruited. The patients were randomly allocated into three groups according to their creatinine clearance rate and were supplemented with daily Se 200 mcg for three months. Plasma Se concentration and RBC GSH-Pxs activity were measured in each patient at the beginning and at the end of the study. This clinical trial was registered in the Iranian Registry of Clinical Trials (www.irct.ir) with registration number ID of IRCT201305318501N2.

**Results:**

Plasma Se concentration and RBC GSH-Pxs activity increased significantly in all three groups of patients with CKD (P < 0.05). There were no significant differences between three groups regarding baseline plasma Se (P = 0.268) and RBC GSH-Pxs activity (P = 0.741).

**Conclusions:**

Se supplementation can increase plasma Se concentration and RBC GSH-Pxs activity in patients with different stages of CKD.

## 1. Background

Chronic kidney disease (CKD) has a high worldwide prevalence and is an important cause of morbidity and mortality due to cardiovascular diseases, malignancies, and others serious complications (1). Many studies have shown increased oxidative stress in patients with CKD (2). Oxidative stress is one of the etiologic factors in several chronic diseases including atherosclerosis and cancer (3). There are increasing evidences of some essential trace elements deficiency in patients with CKD (4). Selenium (Se) and other trace elements such as zinc and copper play an important role in biological defense against oxidative stress (5).

As a trace element, Se is a key component of several enzymes such as glutathione peroxidase (GSH-Pxs) that catalyze reduction of hydrogen peroxide (H_2_O_2_) and other organic hydroperoxides to water through glutathione as the reducing agent (6). Therefore, GSH-Pxs protects cell membrane lipids, proteins, and DNA against oxidative stress (7). In healthy individuals, plasma GSH-Pxs is synthesized mostly by the kidneys (8). Some studies showed that Se administration had no effect on plasma GSH-Pxs activity in patients with CKD (9), while others found increased activity of this enzyme after Se supplementation in these patients (10).

## 2. Objectives

The aim of our study was to determine the effect of Se supplementation RBC GSH-Pxs activity in patients with different stages of CKD.

## 3. Patients and Methods

### 3.1. Patients Population

This study was a randomized clinical trial conducted in Tooba Clinic of Mazandaran University of Medical Sciences (Sari, Iran) from December 2011 to May 2013. An open label prospective study of three months duration was performed in 45 out of 121 patients with CKD who attended the clinic. Inclusion criteria were as follows: patient age between 20 to 65 year, creatinine (Cr) clearance of 15 to 89 mL/min, and diagnosed as CKD for at least six months. The patients were excluded if they had any of the conditions known for interference with antioxidant system such as acute infection, acute myocardial infarction in recent six weeks, active liver disease, treatment with antioxidant drugs (such as vitamins C and E), hemodialysis, and active smoking. This study was approved by the Ethics Committee of Mazandaran University of Medical Sciences and all patients signed a written informed consent before the beginning of the study.

The patients were randomly allocated into three groups in a 1:1:1 ratio (with 15 patients in each group) according to the stage of CKD:

1. mild (Cr clearance, 60-89 mL/min);
2. moderate (Cr clearance, 30-59 mL/min); and
3. severe (Cr clearance, 15-29 mL/min).

All patients were supplemented with one tablet of Se 200 mcg (Nature Made, USA) daily for three months.

### 3.2. Data Collection

After overnight fasting, blood samples were drawn from all patients into tubes containing lithium heparin at the beginning of the study and after three months of Se supplementation. Whole blood was separated into RBCs and plasma by centrifugation (40℃, 5000 rpm, ten minutes). Plasma Se concentration was analyzed by graphite electrothermal atomic absorption spectrometry using an AA240FS apparatus (Varian model). The RBC GSH-Pxs activity was assessed by sandwich ELISA kit (UK, 2012) using tertbutyl hydroperoxide as a substrate. One unit (U) of the enzyme activity was determined as one mol NADPH oxidized per minute per one gram hemoglobin (U/gHb). Serum Cr and urea levels were measured with (Pars Azmoon kit made in Iran), using the Auto-analyzer BT-3000 (made in Italy). Cr clearance was determined by the Cockcroft–Gault formula (11).

### 3.3. Statistical Analysis

Data was analyzed by SPSS version 17.0 (SPSS Inc., Chicago, Illinois, USA). Continuous variables were reported as the mean ± standard deviation. Chi-square and paired samples t-test were used to evaluate qualitative and quantitative parameters, respectively. P value < 0.05 was considered as statistically significant.

## 4. Results

Mean age of all patients in three groups was 54 ± 11 year and male to female ratio was 24:21. Clinical and laboratory characteristics of patients in each group are summarized in Table 1. Table 2 compares mean plasma concentration of Se and GSH-Pxs activity at the baseline and after three months in each group. There were significant increase of plasma Se and RBC GSH-Pxs activity in all three groups after Se supplementation (P < 0.05). However, there were not any significant differences between three groups with regard to the baseline plasma Se (P = 0.268) and RBC GSH-Pxs activity (P = 0.741).

**Table 1.** Basic Characteristics of Patients With Chronic Kidney Disease ^a, b^

	Group I (Mild) (n = 15)	Group II (Moderate) (n = 15)	Group III (Severe) (n = 15)	P value
**Age**	50.80 ± 8.35	56.80 ± 8.72	55.52 ± 14.95	0.45
**Sex (male to female ratio)**	8:7	7:8	9:6	0.87
**Plasma Cr, mg/dL**	1.58 ± 0.13	2.34 ± 0.25	3.31 ± 2.33	0.001
**Cr clearance, mL/min**	76.40 ± 3.92	43.40 ± 4.78	21.10 ± 5.5	0.001

^a^ Data are presented as mean ± SD.

^b^ Abbreviation: Cr, Creatinine.

**Table 2.** Plasma Selenium Concentration and Glutathione Peroxidase Activity at the Baseline and at the Three-Month Endpoint ^a, b^

Group	Plasma Se, ng/mL			Red Blood Cells GSH-Pxs Activity, U/gHb		
	Baseline	Endpoint	P Value	Baseline	Endpoint	P value
**Group I (mild) (n = 15)**	44.16 ± 6.72	76.71 ± 8.91	0.001	20.18 ± 9.31	25.47 ± 7.83	0.001
**Group II (moderate) (n = 15)**	38.23 ± 5.61	67.52 ± 7.50	0.001	16.91 ± 6.96	23.08 ± 7.03	0.001
**Group III (severe) (n = 15)**	41.52 ± 3.17	56.17 ± 6.61	0.02	17.18 ± 7.11	22.58 ± 5.31	0.001

^a^ Data are presented as mean ± SD.

^b^ Abbreviation: GSH-Pxs, glutathione peroxidase.

## 5. Discussion

Our study showed that Se supplementation was effective in increasing cellular GSH-Pxs activity and plasma Se level in the patients with different stages of CKD. In CKD, GSH-Pxs plays an important role in protection of cells against oxidative stress (7). Therefore, Se supplementation may increase survival of these patients.

Oxidative stress has been associated with the outcome in patients with CKD (12). Reactive oxygen species production progressively enhances with advancing stage of CKD (13). The degree of oxidative stress has been shown to be influenced by alterations in the plasma level of some trace elements such as Se, copper, and zinc (14, 15). Zachara et al. observed that in patients with CKD, Se supplementation (200 mcg/24 h) for three months caused a significant increase in plasma Se concentration at any stages of disease, whereas plasma GSH-Pxs activity was enhanced only at the mild stage of the disease (16). In another study in Poland, Se supplementation for patients with CKD on hemodialysis had no effect on the plasma GSH-Pxs protein level (9). However, Nishioka et al. reported that Se supplementation might induce GSH-Pxs synthesis in the extrarenal tissues and suggested that patients with CKD had to be given Se in the early stage of disease (17). Salehi et al. also showed that selenium was an effective supplement for reducing of malnutrition in patients on hemodialysis through alleviating oxidative stress and inflammation (18). It has been shown that renal proximal tubular epithelial cells have high concentration of GSH-Pxs; therefore, this enzyme may have a specific antioxidant role in the kidney (19). In contrast to differences in RBC GSH-Pxs activity, a progressive decline in plasma GSH-Pxs activity in patients with CKD (20) is related to the fact that kidneys are the main source of this enzyme in the body.

Our study had some limitations. First, we did not compare plasma Se concentration and RBC GSH-Pxs activity in patients with CKD with healthy control group. However, others have shown that the levels of these parameters are lower in patients with CKD than in healthy individuals (16, 21). Second, we conducted this study in a single center with small number of patients. Further multicenter clinical trials are required to determine exact effect of Se supplementation on antioxidant system of the patients with CKD. In conclusion, this study suggested that Se supplementation was effective to improve cellular GSH-Pxs activity in the patients with different stages of CKD.
